# Accessory breast cancer in the inframammary region: a case report and review of the literature

**DOI:** 10.1186/s40792-021-01285-6

**Published:** 2021-09-08

**Authors:** Yuka Eguchi, Heiji Yoshinaka, Naoki Hayashi, Kazunobu Sueyoshi, Keiichiro Uchikura, Yuki Nomoto, Ayako Nagata, Hazuki Saho, Yoshiaki Shinden, Takao Ohtsuka

**Affiliations:** 1 Department of Breast Surgery, Kagoshima City Hospital, 37-1 Uearata-chou, Kagoshima-shi, Kagoshima-ken 890-8760 Japan; 2 Department of Pathology, Kagoshima City Hospital, Kagoshima-shi, Japan; 3 Uchikura Clinic, Aira-shi, Japan; 4 Department of Digestive, Breast and Thyroid Surgery, Kagoshima University Hospital, Kagoshima-shi, Japan

**Keywords:** Accessory breast cancer, Inframammary region, Sentinel node biopsy

## Abstract

**Background:**

Although a few cases of accessory breast cancer (ABC) have been reported, most were in the axillary region. We encountered an extremely rare case of ABC in the inframammary region (IMR).

**Case presentation:**

The patient was a 68-year-old postmenopausal woman who had noticed a congenital accessory nipple in her left IMR with slight, occasional discharge 20 years ago. Recently, she noticed a mass under the accessory nipple and visited a nearby clinic; fine-needle aspiration cytology of the mass revealed that it was malignant. She presented to our department 2 weeks after she had noticed the mass. Physical and imaging examinations showed an irregular tumor mass 1.7 × 1.4 × 1.0 cm in size connected to the accessory nipple beneath the left normal breast. Neither distant metastasis nor lymph node swelling was observed. Ultrasound-guided core needle biopsy revealed the mass to be invasive ductal carcinoma. We diagnosed her tumor as ABC in the left IMR; cT1cN0M0: stage IA. Curative wide resection with sentinel node biopsy was performed. Intraoperative evaluation of the frozen section revealed a hot and green ipsilateral axillary lymph node that was free from carcinoma; therefore, nodal dissection was avoided. Histopathological examination including immunochemical staining revealed that the tumor was invasive ductal carcinoma arising from the accessory breast tissue, scirrhous type, 1.7 × 1.4 × 1.0 cm in size, with a solid intraductal component. There was no lymphovascular infiltration, and the surgical margin was 1.5 cm or more. The tumor was estrogen and progesterone receptor-positive, Her2/neu-negative, and had a Ki-67 labeling index of 20%. There was no involvement of the three hot and/or green nodes. The final classification was pT1cN0(sn)M0: stage IA. Letrozole 2.5 mg/day will be administered for 5 years as adjuvant hormonal therapy.

**Conclusions:**

A cutaneous and/or subcutaneous lesion except for proper breast tissue on the milk line, or mammary ridge from axilla to groin may be an accessory breast tissue. Its serial abnormalities must be worried malignant potential to ductal carcinoma which needs some imaging and pathological examinations for definitive diagnosis and appropriate treatment according to the usual orthotopic breast cancer without delay.

## Background

Although a few cases of accessory breast cancer (ABC) have been reported, most were in the axillary region. Although ectopic nipples may sometimes be seen in the inframammary region (IMR), most are polythelia without ductal tissue; therefore, malignant changes are infrequent. We encountered an extremely rare case of ABC in IMR. The cancer developed from the congenital accessory breast tissue (ABT); therefore, clinical and pathological diagnosis and staging revealed that the cancer was invasive ductal carcinoma, cT1cN0M0, Stage IA. Curative wide resection and sentinel lymph node biopsy (SNB) were performed immediately.

## Case presentation

The patient was a 68-year-old postmenopausal woman. Since birth, she had had an accessory nipple below her left normal breast. Since the past 20 years, she occasionally experienced a slightly bloody discharge from the accessory nipple, but cytologic examination revealed no malignancy.

In March 2020, she noticed a slightly tender mass without any discharge under the accessory nipple. She visited a nearby clinic and underwent fine-needle aspiration cytology. She presented to our department 2 weeks after she had noticed the mass because cytology showed that the mass was malignant.

**Fig. 1:**
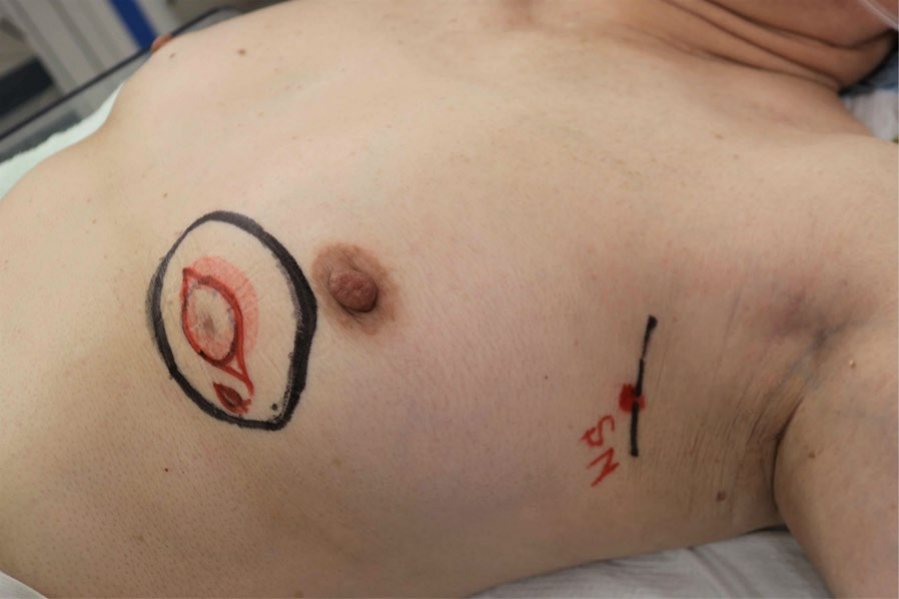
Wide resection (black circle) and sentinel node biopsy (SN)

**Fig. 2:**
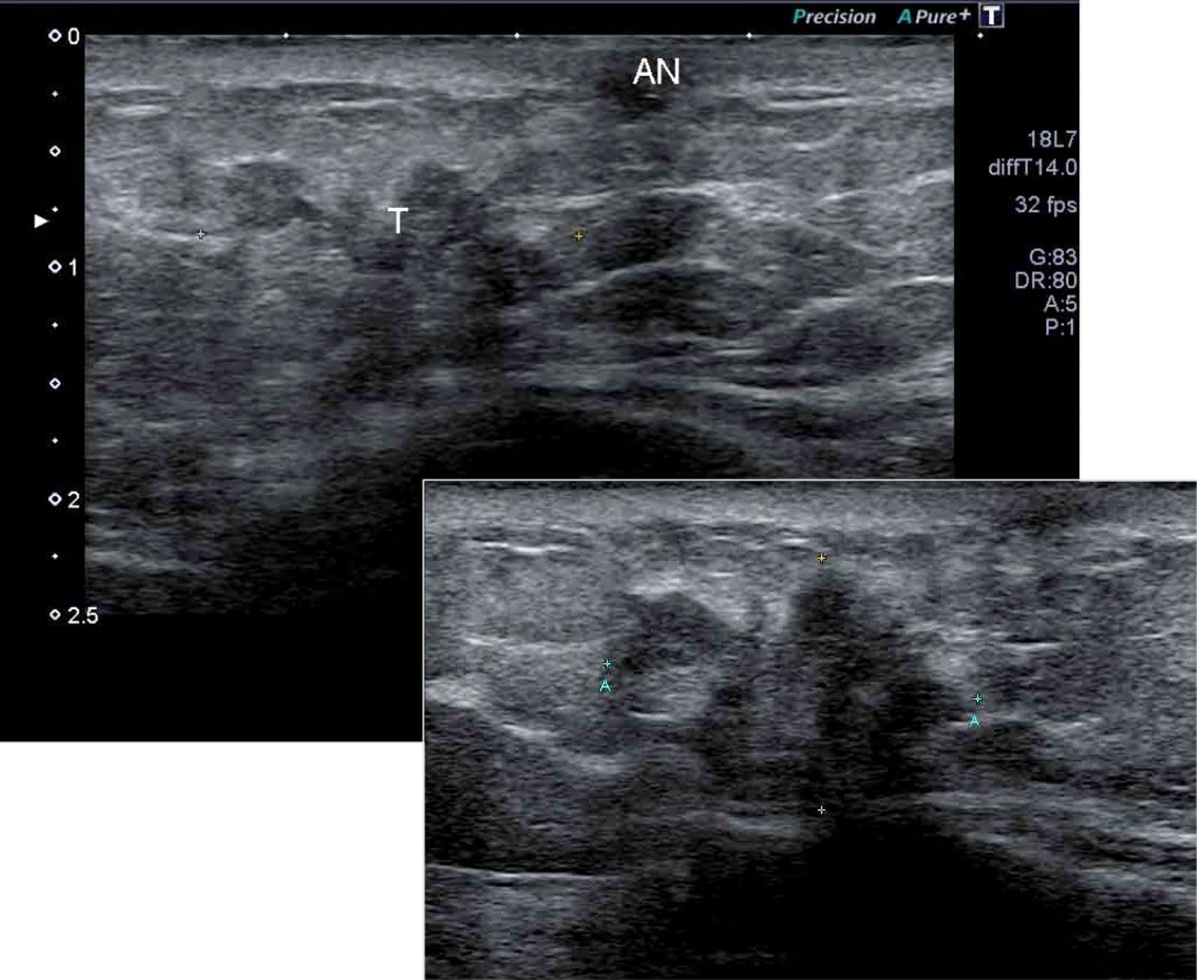
A hypoechoic mass 1.7 × 1.4 × 1.2 cm in size (T) under the inner site of the accessory nipple (AN)

**Fig. 3:**
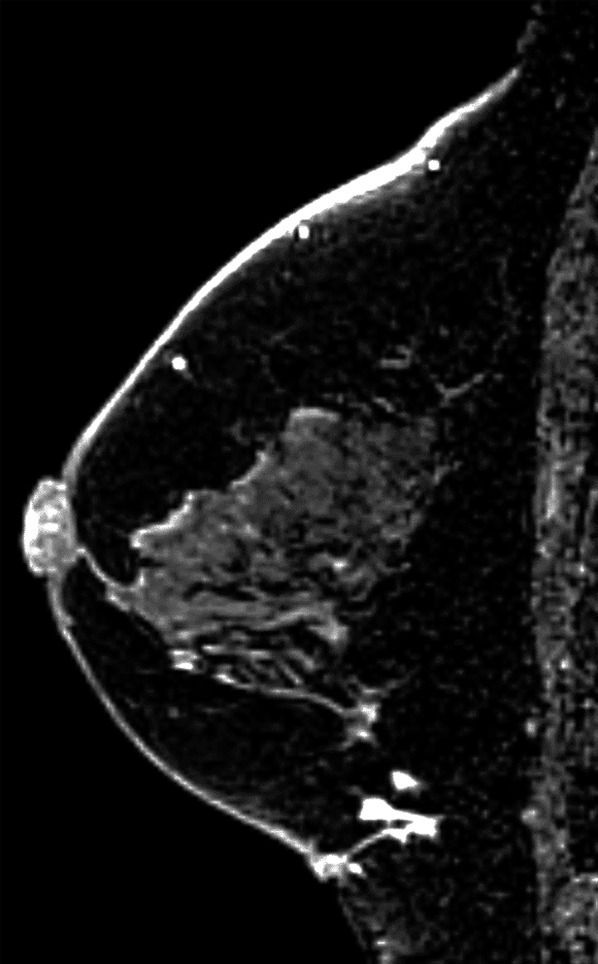
Enhanced irregular mass connected to the dermal nodule

On physical examination, an adzuki bean-sized accessory nipple that was pale pink in tone and with a narrow areola was seen in the IMR below her left normal breast, and a hard mass was palpable beneath it (Fig. 1). Mammography, ultrasonography (US), and magnetic resonance imaging (MRI) showed an irregular and heterogeneous mass with an unclear margin approximately 1.7 × 1.4 × 1.0 cm in size below her left normal gland. The mass appeared to be connected to the dermal nodule, suggesting an accessory nipple, and was clearly visible in contrast-enhanced MRI (Figs. 2, 3). Computed tomography, bone scintigraphy, and US showed neither distant nor nodal metastasis.

US-guided core needle biopsy revealed the mass to be an invasive ductal carcinoma. We diagnosed her tumor as ABC in the left IMR (cT1cN0M0, stage IA).

**Fig. 4:**
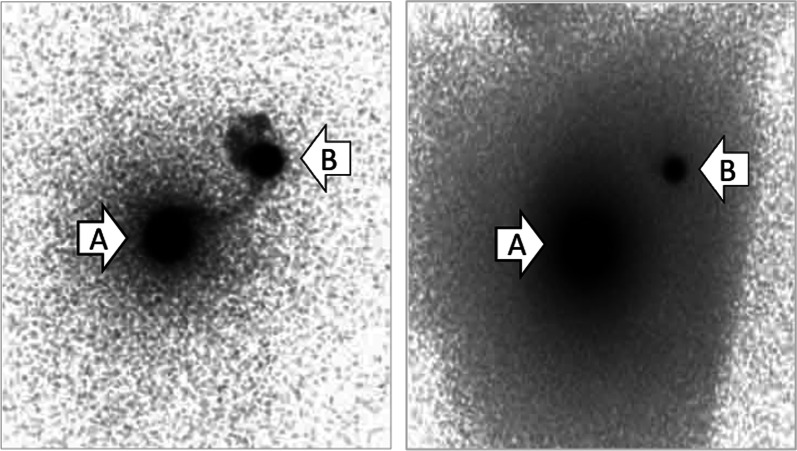
Both RI and ICG were detected in an axillary node. (RI scintigraphy, **A** Injection Site, **B** Sentinel Node)

One month after her first visit, she underwent wide resection and SNB. According to the map drawn with US guidance, the mass and surrounding adipose tissue and skin, including the accessory nipple and areola, were removed en bloc with 1–2 cm of horizontal margin around the entire circumference (Fig. 1). For SNB, technetium-99 m phytate 74 MBq radioisotope (RI) was intradermally injected into the left normal areola on the day before surgery, and indocyanine green (ICG) 5 mg was injected into the skin at the side of the accessory nipple just before surgery. We used a gamma ray-detecting probe and a fluorescence ICG detector to detect and remove three axillary nodes that contained RI and/or ICG. One node that contained both RI and ICG was diagnosed as free from carcinoma by frozen section procedure, and the other two nodes that included ICG were diagnosed as permanent section procedures (Fig. 4).

**Fig.5:**
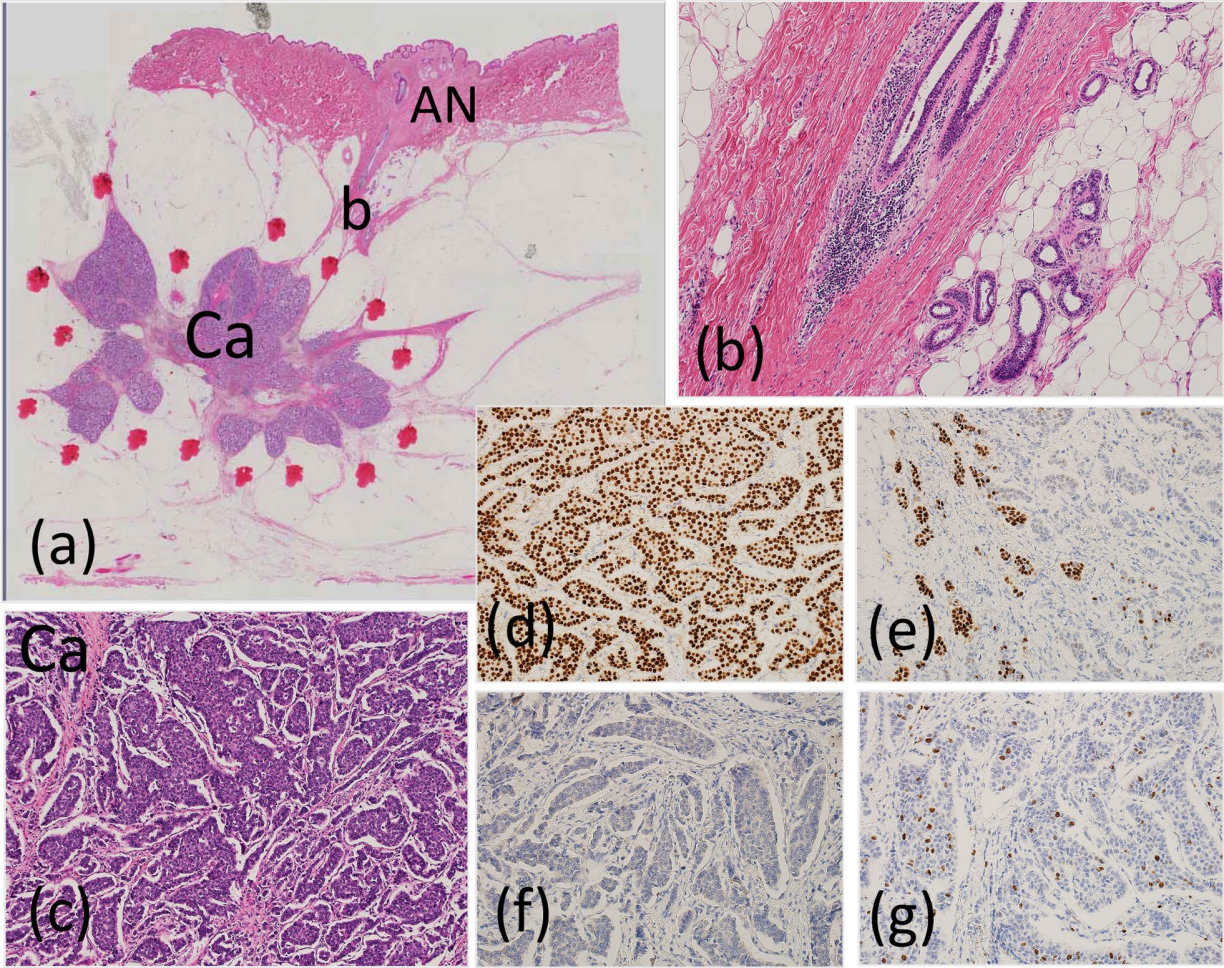
**a** Invasive ductal carcinoma including an intraductal component was connected to the accessory nipple (AN) with normal ductal tissue. **b** Normal ductal tissue under the AN. **c** Invasive ductal carcinoma × 400. **d** Estrogen receptor-positive. **e** Progesterone receptor-positive. **f** Her2/neu-negative. **g** Ki-67 labeling index, 20%

Histopathological examination including immunochemical staining revealed that the tumor was an invasive ductal carcinoma (pT1cN0(sn)M0, stage IA) arising from the ABT, scirrhous type, and 1.7 × 1.4 × 1.0 cm in size, including the intraductal component. Its invasive size was 1.4 cm in diameter, there was no lymphovascular infiltration, and the surgical margin was 1.5 cm or more. The tumor was estrogen and progesterone receptor-positive and Her2/neu-negative and had a Ki-67 labeling index of 20%. None of the three resected nodes showed involvement. The carcinoma was located just beneath the accessory nipple and was connected to it by the usual mammary ducts (Fig. 5). Eight months after surgery, the patient is well. Letrozole 2.5 mg per day will be administered for 5 years as adjuvant hormonal therapy.

## Discussion

Although a few cases of ABC have been reported, most of them were located in the axillary region [1, 2]. Our case is of great interest for several reasons: (1) ABC in the IMR is extremely rare [1, 3]; (2) complete ABT consisting of the nipple, areola, and glandular tissue corresponding to Kajava’s Class I had been previously recognized because of congenital appearance and nipple discharge; (3) pathological diagnosis of the invasive ductal carcinoma and clinical staging of cT1N0M0: Stage IA were properly performed before surgery; and (4) sentinel nodes could be detected and confirmed to be free from involvement by a RI/ICG-fluorescence double method so that nodal staging was assessed and clearance could be avoided.

Ectopic breast tissue has two forms: supernumerary and aberrant. A small minority of ectopic breast tissues are aberrant; they are most commonly found in the axilla. Ectopic breast tissue consists of an isolated fragment of glandular tissue without any connection between the inside and outside and is more prone to malignant transformation than normal breast tissue or ABT because stagnation arising in the ductal lumens promotes the development of malignancy [1]. A majority of ectopic breast tissues are supernumerary; they have been recently termed as “accessory” breast tissue. The most common form of ABT is polythelia, in which ductal tissue is absent. This condition is present in 2–5% of the general population [4]. Over a century ago, Williams, Iwai, and Kajava described ABT in detail. In 1891, Williams presented a diagram showing the mammary arrangement of early human progenitors, who had at least seven pairs of breasts on the ventral aspect of the trunk. His diagram is nearly identical to the milk line or mammary ridge along which later researchers usually located ABT [1, 2, 5, 6]. Our case corresponds to the fifth pair in his diagram, i.e., just below and slightly medial to the normal female breast. Williams stated that more than three-fourths of all instances of supernumerary mammary structures had been found in this position. With regard to the development of cancer in ectopic breast tissues, Williams found that 13 (9.8%) of 132 cases of cancer of the mammary region in women had originated in supernumerary mammary structures that were outside the normal breasts. However, none of the 13 cases had cancer that originated in the fifth pair in his diagram [7]. In 1907, Iwai reported in the *Lancet* that the incidence of polymastia in a hospitalized Japanese population was 2.04 or 3.7%, 5.19 or 6.0% for women and 1.68 or 2.04% for men [8–11]. In 1915, Kajava classified supernumerary nipples into 6–13 types according to the existence of glandular tissue, nipple, areola, skin, and patch of hair and further divided them into two categories: polymastia with glandular tissue and polythelia without glandular tissue [2, 8, 12]. His classification is still used today because glandular tissue is subject to hormonal effects and medical conditions such as enlargement, swelling, tenderness, lactation during puberty, premenstruation and/or pregnancy, as well as development of fibroadenoma, adenoma, cysts, abscesses, mastitis, and particularly ductal carcinoma. Our case corresponds to Kajava’s class I; complete breast with nipple, areola, and glandular breast tissue all in place (also known as polymastia) [1, 5, 8, 12, 13].

The prevalence of ABT varies greatly, ranging from 0.22 to 6% of the population [14]. It varies according to ethnicity, sex, geographic region, and the method used to determine the presence of ABT. East Asian populations, including Japanese, are believed to have a higher incidence of ABT than Caucasians [5, 6, 8, 14].

A malignancy arising from ABT is a very rare condition; the incidence is approximately 6% because the most common form of ABT is polythelia without glandular tissue, and there is no convincing evidence that the glandular tissue of polymastia is more prone to malignant change than normal breast parenchyma [4, 5, 12]. The incidence of ectopic breast cancer, including the aberrant type, is low, i.e., 0.2–0.6% of all breast cancers. The most common location is the axilla (60–70%), although it can develop in other less common locations such as IMR (5–10%) and rarely in the thighs, perineum, groin, and vulva [3, 13, 15]. Of the 1,148 cases of primary breast cancer we encountered from 1992 to 2020, only five (0.44%) were ABC, and all four previous cases were in the axilla.

We found six case reports of ABC in the inframammary or upper abdominal region [1, 3, 6, 16–18]. Madej et al. reported a case of advanced cancer (pT4N1M0: stage IIIB) [16, 19]. The patient reported by Broker et al. had swelling near an accessory nipple. Physical examination and X-ray examination indicated a benign tumor, but excision and pathological examination revealed that it was breast carcinoma. After follow-up examination, re-excision with a sentinel node procedure was performed. Finally, the patient was found to have a pT1bN0(SN)M0; stage IA breast cancer in the ABT [17, 19]. The case reported by Francone et al. resembled ours, although the cancer was more advanced (pT1cN1aM0: stage IIA) [1, 19]. Wysokinska reported an early stage, mainly in situ ectopic breast cancer occurred in a patient with prior history of the left breast cancer treated with modified radical mastectomy. Ectopic breast cancer was found 20 years after left mastectomy because of about two weeks of pain associated with a small lump located on the right, i.e. contralateral anterior chest on the lower aspect of the rib cage, 2 cm below proper breast [6]. In the case reported by Randy et al., ABT was located in the left upper abdomen, and the patient’s mammogram seemed to correspond to the fifth or sixth pair of William’s diagram; the final diagnosis was inflammatory breast cancer [7, 18]. In Thasanabanchong and Vongsaisuwon’s recent case, the patient presented with a subcutaneous mass over the costal ridge. No malignancy was suspected when the mass was excised, but the pathological examination showed that the tissue was malignant. Therefore, nodal metastasis had to be considered, and SNB was performed 7 days after excision. This case had an early stage (pT1cN0M0: stage IA), similar to our case [3, 19].

SNB can be used for lymph node staging of ABC just as for orthotopic breast cancer [1, 3, 13, 15–18, 20, 21]. The classical Sappey’s line serves as a “watershed,” defined as an imaginary line transversely connecting the umbilicus to the L2 on the back, separating the area of lymphatic drainage of the torso. The lymphatic drainage of the skin and subcutaneous tissue above Sappey’s line is believed to drain into the ipsilateral axillary nodes, whereas that below the line drains into the ipsilateral groin nodes [3, 17, 18]. As in our case, the ipsilateral axillary nodes were sentinel nodes in five of six previously reported cases of ABC in IMR. In two of the five cases, radical axillary lymphadenectomy was performed because metastases to the sentinel nodes were found. In contrast, ABC with SNB in the vulva was reported by Intra [21] and Ishigaki [15]. In their cases, ipsilateral inguinal sentinel nodes were found to be free from involvement during surgery, and inguinal lymph node dissection was avoided.

Although the injection site of the RI/ICG was not described in the four of five cases of ABC in the IMR, it was probably intradermal by the side of the tumor modeled after SNB of the melanomas. In Thasanabanchong and Vongsaisuwon’s case, RI was injected into the ipsilateral periareolar area and the lesion site where the excision was performed [1, 3, 16, 18]. We used the RI/ICG fluorescence double method to improve accuracy. RI was injected intradermally into the ipsilateral normal areola, where lymphatic vessels are rich, and ICG was injected into the skin by the side of the accessory nipple.

## Conclusions

A cutaneous and/or subcutaneous lesion on the milk line or mammary ridge from the axilla to the groin may be ABT. Abnormalities of this tissue are concerning because of the malignant potential for ductal carcinoma, requiring imaging and pathological examinations for definitive diagnosis and appropriate prompt treatment that is in accord with the treatment of usual orthotopic breast cancer.

## Data Availability

None.

## Abbreviations

ABC: Accessory breast cancer
IMR: Inframammary region
ABT: Accessory breast tissue
SNB: Sentinel node biopsy
US: Ultrasonography
MRI: Magnetic resonance imaging
RI: Radioisotope
ICG: Indocyanine green
